# The secrets of El Dorado viewed through a microbial perspective

**DOI:** 10.3389/fmicb.2012.00239

**Published:** 2012-07-10

**Authors:** Aurelio M. Briones

**Affiliations:** Division of Soil and Land Resources, Department of Plant, Soil and Entomological Sciences, University of Idaho,Moscow, ID, USA

**Keywords:** Amazon Dark Earths, *terra preta*, biochar, charcoal, extracellular electron transfer, soil microbial fuel cells, Terra preta, biochar, extracellular electron transfer

## Abstract

The formation of the Amazon Dark Earths was a model of sustainable soil management that involved intensive composting and charcoal (biochar) application. Biochar has been the focus of increasing research attention for carbon sequestration, although the role of compost or humic substances (HS) as they interact with biochar has not been much studied. We provide a perspective that biochar and HS may facilitate extracellular electron transfer (EET) reactions in soil, which occurs under similar conditions that generate the greenhouse gases methane and nitrous oxide. Facilitating EET may constitute a viable strategy to mitigate greenhouse gas emission. In general, we lack knowledge in the mechanisms that link the surface chemical characteristics of biochar to the physiology of microorganisms that are involved in various soil processes including those that influence soil organic matter dynamics and methane and nitrous oxide emissions. Most studies view biochar as a mostly inert microbial substrate that offers little other than a high sorptive surface area. Synergism between biochar and HS resulting in enhanced EET provides a mechanism to link electrochemical properties of these materials to microbial processes in sustainable soils.

## INTRODUCTION

### THE SEARCH FOR EL DORADO

Microbes have played prominent roles in the history of ancient American civilizations. To the Spanish conquistadors, they became unwitting allies – smallpox, typhus, diphtheria, measles – these and other diseases contributed to the deaths of perhaps 95% of the indigenous people in the Americas within 130 years after first contact with Europeans (Dobyns and Swagerty, 1983). Within a few decades after first contact, the desire for riches lured some conquistadors deep into the Amazon jungle in pursuit of one of the most persistent myths of wealth in the Americas: “El Dorado,” the legendary Lost City of Gold. It was in search of El Dorado that brought Francisco Orellana through the jungles of the Amazon – he would later become known as the first European to successfully navigate the Amazon River. It was after this arduous journey that Orellana (or more accurately, his chronicler Gaspar de Carvajal) reported densely populated settlements within the Amazon. Until recently, such tales were considered fanciful since permanent settlements in this tropical rainforest were regarded as an impossibility. The lush green of the jungle is deceiving; the soils of the Amazon are too poor to support any kind of intensive agriculture and without this as foundation, no sophisticated civilization could thrive (Meggers, 1971). However, recent archeological evidence suggest otherwise (Mann, 2005; Glaser, 2007; Heckenberger, 2009). It is now becoming clear that settled communities existed throughout the Amazon basin during pre-Columbian times, and wherever there are traces of ancient habitation, not far will be found dark colored soils known locally as *terra preta de Indio* (Indian dark earth; also referred to as Amazon Dark Earth, ADE). Sadly, the original populace that created *terra preta* also became victim to the onslaught of disease after first contact. However, their legacy in the form of the land they managed lives on.

### TERRA PRETA

*Terra preta* was first described by modern soil science in the mid-1960s (Sombroek, 1966). One of the distinguishing features of ADE is the high charcoal or black carbon content, about 70% higher compared to the adjacent non-ADE soils. These soils are highly fertile in comparison to the poor and highly weathered surrounding (parent) soil. Aside from elevated levels of black carbon, ADE is characterized by higher organic C, N, Ca, and P, higher cation exchange capacity, higher pH, and higher base saturation than the surrounding soils (Glaser et al., 2001; Lehmann et al., 2003). While nutrient poor, the parent soils that host ADEs are rich in iron and aluminum oxides (Glaser et al., 2002). The most intriguing property of ADE soils is its capacity to sustain its fertility even without applying mineral fertilizers (Madari et al., 2004), a feat that modern agriculture has not been able to accomplish. Charcoal in soil (now referred to as biochar, to differentiate it from charcoal for heating) is persistent: its polycyclic aromatic structure resists microbial degradation and lasts in soil for millenia (Kuzyakov et al., 2009). Thus when charcoal is applied to soil, C is sequestered for a long period of time, making this practice essentially carbon negative (Glaser et al., 2009) and therefore attractive as a means to mitigate climate change while improving soil health.

**FIGURE 1:**
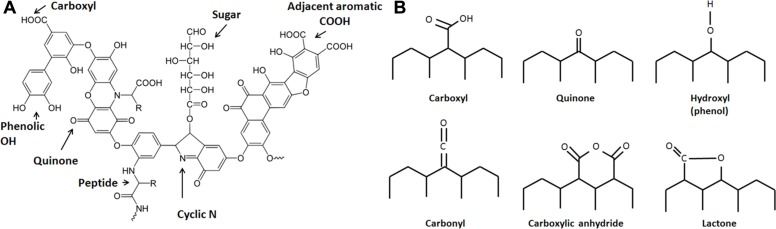
Structures of **(A)** a model humic acid (adapted from Stevenson, 1994) and **(B)** surface functional groups on activated carbon, which are expected to be similar to those on biochar. These groups are bonded to aromatic rings (adapted from Yang, 2003).

Evidence suggests that aside from incorporating charcoal into the soil, the Amazonians practiced intensive soil composting, essentially creating composting fields that today bear evidence of widespread incorporation of organic wastes including mammalian and fish bones and feces (Lima et al., 2002; Schaefer et al., 2004; Birk et al., 2011). Therefore, the ADE soils are the product of intensive soil management that involved both charcoal amendment and composting, representing inputs of both highly recalcitrant and relatively labile forms of organic matter. Co-application of char and compost during the creation of ADE soils is a model of sustainable soil management. Since the labile fractions of soil amendments are rapidly mineralized, the components that elicit long-term effects are the recalcitrant fractions, mainly charcoal and humic substances (HS) from compost. HS (**Figure 1A**) are extremely complex and heterogeneous high molecular weight organic materials that often contain polyaromatic structures that include oxygen-containing functional groups, including quinone moieties. Humus is commonly viewed as an essential component of rich soil, although stable humus no longer contributes nutrients and is commonly viewed as important mainly to improve the physical structure of soil. However, recent findings suggest that dissolved HS are redox-active and contribute to anaerobic microbial metabolism by mediating extracellular electron transfer (EET), in effect shuttling electrons between metal-respiring bacteria and metal oxides (Lovley et al., 1996). Quinone moieties within functional groups of HS have been implicated as responsible for this type of electron transfer reaction (Lovley et al., 1996; Scott et al., 1998; Stack et al., 2004; Roden et al., 2010).

### INFLUENCE OF CHAR AND COMPOST ON EET IN SOIL

The paradigm of microbial respiration involves soluble compounds such as oxygen that acts as a terminal electron acceptor. Microbial EET occurs when solid substances such as metal oxides or electrode materials are used as electron acceptors during respiration (Hernandez and Newman, 2001). Because microbes that use electrode materials as electron acceptors form biofilms on the electrode surface, these systems, called microbial fuel cells (MFCs), are well-suited to study the physiology of microbial EET. When a microbe uses an anode as an electron acceptor, it conserves energy by oxidizing a substrate (which may be organic or inorganic, such as acetate or hydrogen) and transferring the resulting electrons to the exogenously provided anode. This requires the absence of oxygen which is more energetically favorable as an electron acceptor. The transfer of electrons to a solid acceptor such as an anode may occur *via* intrinsic microbial mechanisms such as those involving direct cell contact to the solid electron acceptor (through the use of cell surface proteins or structures such as specialized pili); through microbially synthesized electron shuttles (Lovley and Nevin, 2008; Marsili et al., 2008; von Canstein et al., 2008); and through various natural substances such as HS and plant exudates (Lovley et al., 1996; Nevin and Lovley, 2000; Hernandez et al., 2004) that shuttle electrons to solid electron acceptors such as Fe(III) oxides. Lovley and colleagues in 1996 first showed that *Geobacter metallireducens* is able to utilize humic acids as electron acceptors using acetate as a substrate (Lovley et al., 1996). The microorganism is required to reduce the humics although in the absence of the microorganism, Fe(III) reduction by reduced HS proceeds, proving that HS act as an electron shuttle. This implies that no contact between the microorganism and a solid electron acceptor is needed in the presence of electron shuttles such as HS. Under conditions of high humus loads, such as in composted wet soil, both soluble and solid phase HS may mediate electron transfer to metal oxides (Roden et al., 2010). Since mature compost is essentially humified organic matter, we would expect this material to have a higher capacity for mediating EET compared to the uncomposted, raw organic material – this has been confirmed in experiments comparing EET capacity between sewage sludge and composted sludge (Huang et al., 2010).

The electrical properties of charcoal have long been recognized (Ford and Greenhalgh, 1970; Coutinho and Luengo, 1993; Mochidzuki et al., 2003). Carbon-based materials, including activated carbon, are frequently used as electrode material in MFCs (Logan, 2008; Jiang and Li, 2009; Zhang et al., 2009; Zhu et al., 2011). Pyrolyzed carbon shares similar characteristics with HS including the presence of surface active groups including quinone moieties within polyaromatic structures (Matsumura and Takahashi, 1979; **Figure 1B**). Charcoal itself is a source of humic acids that are formed during pyrolysis (Trompowsky et al., 2005) or after sufficient aging (Nishimura et al., 2006), which is probably a consequence of a slow oxidative process. Viewing charcoal as a material that mediates extracellular electron transport is mostly unexplored in the field of biochar (soil amendment) research. To enhance the power generation of MFCs, activated carbon may be treated with ammonia (Cheng and Logan, 2007), nitric acid, and ethylenediamine (Zhu et al., 2011) to significantly increase the power output of MFCs. These treatments most likely change the surface functional groups on activated carbon, increasing groups such as lactam, imide, amide, and ammonium nitrate, which are hypothesized to facilitate the adhesion of microbes that perform EET (Zhu et al., 2011). Since biochar can be manufactured from a variety of biomass sources (including solid organic wastes), the chemical composition of feedstocks should influence their electrochemical characteristics and should be taken into consideration when designing soil amendments.

**FIGURE 2:**
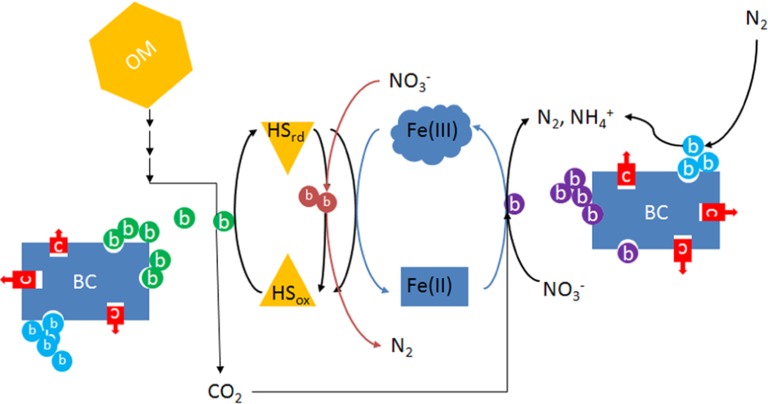
**Diagram illustrating the possible interactions between microbes, biochar, humic substances, and metals in water-saturated soil or sediments**. Because of its abundance, only iron is considered here, although numerous metals may undergo similar processes. OM, organic matter; BC, biochar; HS_rd_, reduced humic substances; HS_ox_, oxidized humic substances; c (red), inorganic or organic contaminants such as heavy metals or pesticides; b (green), humics-reducing bacteria that may also be Fe-reducing; b (light red), nitrate-dependent humics-oxidizing bacteria; b (purple), chemolithoautotrophic nitrate-respiring iron-oxidizing bacteria; b (light blue), nitrogen-fixing bacteria.

Enhanced EET in soil may facilitate electron flow to electron acceptors other than oxygen. For example, a phylogenetically diverse range of bacteria including members of the alpha-, beta-, gamma-, and delta-Proteobacteria utilize HS as electron donor and nitrate as electron acceptor in forest soil, freshwater and marine sediments (Coates et al., 2002). Similar findings have recently been described in agricultural soils (Van Trump et al., 2011), raising the possibility that anaerobic (nitrate) respiration facilitated by HS is an important process in agricultural soils. In most cases, including those in which a reduced metal is used directly as an electron donor, bacteria that oxidize reduced HS for energy require an organic co-substrate for carbon, frequently reduced fermentation products such as acetate and other volatile fatty acids (Straub et al., 1996; Benz et al., 1998). Therefore, electron flows through fermentation and anaerobic respiration processes are favored in the presence of HS under anaerobic conditions. Compared to heterotrophic processes that utilize the same substrate for electrons and carbon, respiration facilitated by HS will require less organic substrate, resulting in more efficient utilization of soil organic matter. How this contributes to long-term stability of soil organic matter, as has been observed in ADE soils would be an interesting area of research. Such research should take into account the possible synergism between biochar and HS that may be the basis of the long-term success of *terra preta*. One might hypothesize that the stability and high surface area of charcoal provides sites that attract bacteria including those involved in EET, thus serving as a stable platform for biofilm formation that supports electron shuttling between microbes and insoluble electron acceptors such as soil metals (**Figure 2**). In the presence of dissolved HS, these metal oxides in effect become more available for electron transfer reactions due to electron shuttling by HS.

### APPLICATIONS

#### C and N cycling

At least part of biochar effects in soils may be attributed to processes occurring under wet, anaerobic, or anoxic conditions. Such conditions are associated with methane and nitrous oxide emissions even in non-waterlogged agricultural soils (Johnson et al., 2007). Anaerobic respiration mediated by char and humics is expected to preempt methanogenesis, since respiratory metabolism through the sequential reduction of electron acceptors (e.g., nitrate before ferric iron before sulfate) yields more energy compared to methanogenesis. When the conditions for anaerobic respiration are met (adequate redox conditions, electron donor, electron acceptor), respiration will be an energetically competitive process compared to methanogenesis. A number of reports suggest that biochar application reduces methane emissions (Haefele et al., 2011; Karhu et al., 2011; Liu et al., 2011). On the other hand, substances that facilitate anaerobic respiration may enhance the production of nitrous oxide, since this gas is an intermediate or terminal product of denitrification, a process that is enhanced by the presence of HS in various environments (Coates et al., 2002; Van Trump et al., 2011). In this case, the co-application of biochar may provide a means of mitigating nitrous oxide emissions (Bruun et al., 2011; Karhu et al., 2011; Taghizadeh-Toosi, 2011). These reports provide no convincing mechanism to link the surface chemical characteristics of biochar to the physiology of microorganisms that are involved in methane and nitrous oxide emissions. Most studies have examined these questions with the viewpoint of biochar being a mostly inert microbial substrate, with the exception of residual volatile organics that remain after pyrolysis. In cases where biochar has been observed to mitigate methane and nitrous oxide emissions, possible explanations include inhibition of methanogenesis or promotion of methanotrophy (in the case of methane mitigation); or immobilization of N (Rondon et al., 2005) as well as soil hydrology (Yanai et al., 2007; in the case of nitrous oxide mitigation). However, enhancing anaerobic respiratory processes that compete with or provides alternatives to methanogenesis or heterotrophic denitrification would, in theory, mitigate emissions of the greenhouse gases associated with these processes. In support of this concept, HS-mediated denitrification is associated with total reduction of nitrate to dinitrogen gas (in most cases, no nitrous oxides emitted; Coates et al., 2002). It is also shown that respiration through Fe(III) reduction competes effectively with methanogenesis in natural systems like tropical forest soils (Dubinsky et al., 2010). Under controlled experimental conditions, enhanced Fe(III) reduction led to as much as 69% reduction in methane emissions compared to controls in rice (*Oryza sativa*) soils (Huang et al., 2009). Enhancing EET in a MFC setup using rice paddy soil as inoculum led to almost complete suppression of methanogenesis when the circuit between anode and cathode is closed (Ishii et al., 2008a).

#### Plant growth and bioremediation

With the societal and environmental benefits associated with biochar, there is now increasing interest in widespread application. However, since the long-term effects of creating *terra preta* have been studied almost entirely in the tropical setting, there is a need to research long-term effects in other settings. Gradual application will most likely be based on cost-benefit analyses. Similar to the original *terra preta*, the most promising application of this ancient technology will be on marginal or degraded soils, such as those contaminated by organic or inorganic pollutants (e.g., mining-impacted land). These lands are often poorly developed with little or no vegetation. There are numerous studies documenting both beneficial and negative effects of charcoal on plant growth (Atkinson et al., 2010 and references within). However, the significance of microbial EET to plant growth is mostly unexplored. In this regard, it is noteworthy that studies in an MFC inoculated with rice paddy soil enriched for a nitrogen-fixing Rhizobiales bacterium that was shown to be involved in electricity generation on the anode (Ishii et al., 2008b) and utilization of Fe(III) as an electron acceptor during anaerobic respiration (Kodama and Watanabe, 2011). The possible components of the nitrogen fixation machinery that is linked to EET is not yet clear, although it may involve the membrane-bound uptake hydrogenases, which are required by nitrogen-fixing bacteria to recycle wasteful hydrogen produced during this energetically demanding process. Hydrogenases are membrane bound enzymes that are directly coupled to the electron transport system via cytochromes and are hypothesized to play a critical role in electron transfer reactions of bioelectrochemical systems (Rosenbaum et al., 2011). In connection with this, it is interesting to note that biochar has been linked to higher rates of nitrogen fixation (Rondon et al., 2007), increased nodulation (Hely et al., 1957), and enhanced carrying capacity for *Rhizobia* (Kremer and Peterson, 1983; Sparrow and Ham, 1983). Aside from beneficial effects for plant growth, biochar application to contaminated lands has a potentially important role in the remediation, revegetation, and restoration of contaminated soils (Beesley et al., 2011). Much of the research conducted in this area link the high sorptive capacity of biochar to sequestration of organic and inorganic pollutants. Sorption of xenobiotics to biochar has two sides: while this may decrease the bioavailability of toxic chemicals, sorption to surfaces that are unavailable to microorganisms may reduce their biodegradation rates. In the case of pesticides, sorption on biochar would also reduce efficacy, thus potentially resulting in higher application rates to compensate for sorption. These problems may be addressed by using biochars that have been precolonized (e.g., used as compost amendments) with microbes, or using feedstock that are dominated by micropores that are accessible to microbes after pyrolysis. The biodegradation of xenobiotics including how the processes are affected by electrochemical properties and interactions with HS (e.g., HS was recently described to influence the speciation and transformations of mercury in a concentration-dependent manner by Gu et al., 2011) is a promising area of research. Electron flow in wet soils through the network of microbes and their appendages, electron donors, electron acceptors, and electron shuttles is a mechanism that more closely links char and its surface properties to microbial metabolism. As research in this field progresses using the modern tools of science, one might be reminded of that age-old quest for El Dorado – this time however, the quest continues on the path that led to the rediscovery of an ancient technology, a legacy that deserves to be studied well and applied wisely to conserve the true wealth of civilizations: our soil.

## Conflict of Interest Statement

The author declares that the research was conducted in the absence of any commercial or financial relationships that could be construed as a potential conflict of interest.
